# Implementation fidelity of infection prevention practices at Debre Tabor comprehensive specialized hospital, Northwest Ethiopia

**DOI:** 10.1186/s12879-023-08263-3

**Published:** 2023-05-22

**Authors:** Endalkachew Mesfin Gebeyehu, Ayal Debie, Lake Yazachew, Samrawit Mihret Fetene, Kefyalew Amogne Azanaw

**Affiliations:** 1 Department of Health Informatics Technology, Debre Tabor Health Science College, Debre Tabor, Ethiopia; 2 Department of Health Systems and Policy, University of Gondar, Gondar, Ethiopia; 3 Department of Nursing, Debre Tabor Health Science College, Debre Tabor, Ethiopia

**Keywords:** Implementation fidelity, Infection prevention practices, Debre Tabor comprehensive specialized hospital, Ethiopia

## Abstract

**Background:**

Healthcare-Acquired Infections are a major problem in the world and within the healthcare delivery system. An estimated 5–10% and around 25% of hospitalized patients have healthcare-acquired infections in developed and developing countries, respectively. Infection prevention and control programs have proven to be successful in lowering the incidence and spread of infections. Thus, this evaluation aims to evaluate the implementation fidelity of infection prevention practices at Debre Tabor comprehensive specialized hospital in Northwest Ethiopia.

**Methods:**

A facility-based cross-sectional design with a concurrent mixed method was used to evaluate the implementation fidelity of infection prevention practices. A total of 36 indicators were used to measure adherence, participant responsiveness, and facilitation strategy dimensions. A total of 423 clients were administered for an interview, an inventory checklist, a document review, 35 non-participatory observations, and 11 key informant interviews were conducted. A multivariable logistic regression analysis was used to identify factors significantly associated with the satisfaction of clients. The findings were presented using descriptions, tables, and graphs.

**Result:**

The overall implementation fidelity of the infection prevention practices was 61.8%. The dimensions of adherence to infection prevention and control guidelines were 71.4%, participant responsiveness was 60.6%, and facilitation strategy was 48%. In multivariable analysis, ward admission and educational level had a p-value of below 0.05 and were significantly associated with the satisfaction of clients with infection prevention practices at the hospital. The major themes that emerged in qualitative data analysis were healthcare worker-related factors, management-related factors, and patient- and visitor-related factors.

**Conclusion:**

The evaluation result of this study concluded that the overall implementation fidelity of infection prevention practice was judged to be medium and needed improvement. It included dimensions of adherence and participant responsiveness that were rated as medium, as well as a facilitation strategy that was rated as low. Enablers and barriers were thematized into factors related to healthcare providers, management, institutions, and patient and visitor relations.

**Supplementary Information:**

The online version contains supplementary material available at 10.1186/s12879-023-08263-3.

## Background

In healthcare environments, infections can spread from Health Care Providers (HCPs) to patients, from patients to HCPs, from patients to patients, or from HCP to HCP [1]. Healthcare-Acquired Infections (HAI) are a major problem in the world within the healthcare delivery system [2].

The Centers for Disease Control and Prevention (CDC) define “healthcare-acquired infection” as infections acquired while receiving treatment for localized or systemic conditions as a result of adverse reactions to the presence of an infectious agent(s) or its toxin(s) within healthcare settings [3].

According to the National Infection Prevention and Control (IPC) reference manual of Ethiopia, the definition of HAI is an infection that occurs in a patient as a result of care at a healthcare facility or occurs if noticed after 48 h of admission [4].

In 1970, the CDC introduced isolation precautions, incorporating policies and practices to prevent the spread of infection in hospitals. In 1985, universal precaution was introduced, and subsequently, in 1987, body substance isolation was introduced. Then, in 1996, standard precautions and transmission-based precautions were developed by the CDC [5]. In 1996, the CDC coined the term “standard precautions” to describe a standard of care aimed at safeguarding healthcare workers and patients against viruses carried through blood or body fluids. In 2007, this was reaffirmed [6].

**Fig. 1:**
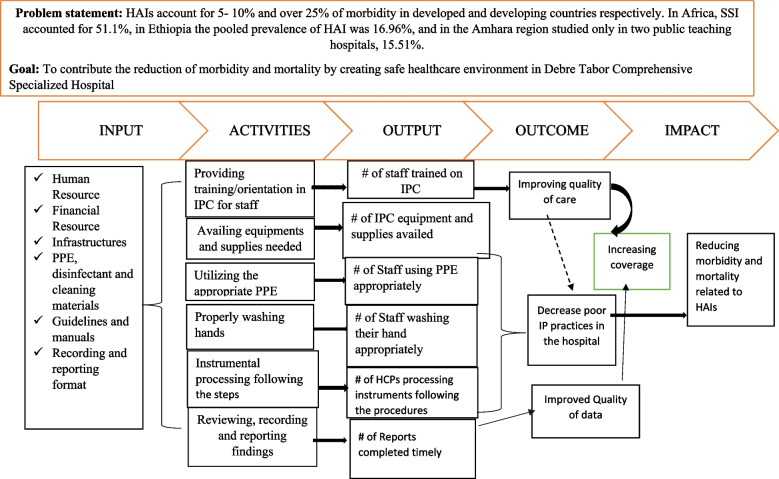
IPC logical model of Debre Tabor comprehensive specialized hospital, 2022

An Infection Prevention and Control (IPC) program was developed by responsible health authorities at different healthcare system levels to reduce the risk of HAIs [7]. As a result, it was incorporated as a critical component of all healthcare systems, affecting patients, visitors, and healthcare providers [8]. The IPC logic model was presented in Fig. 1.

Infection management in hospitals began in the mid-nineteenth century. In the 1950s, however, outbreaks of staphylococcus aureus infection in North America and the United Kingdom promoted the development of contemporary infection control. This program has been part of the hospital’s routine practices since it began 30 years ago [9].

Since around the year 2000, South Africa has had a well-developed national IPC program. Since 2007, the government has been steadily implementing national guidelines on IPC structure, standard and transmission-based measures, and outbreak control measures, with a significant reduction in HAIs [10].

Following the publication of the first national infection prevention guideline, Ethiopia has made significant progress in understanding and implementing evidence-based infection prevention measures in healthcare facilities. However, due to new scientific findings, the recommended evidence-based procedures are constantly changing around the world [11].

Healthcare-acquired infections have a major negative impact on the global healthcare delivery system, resulting in increased morbidity and mortality and excessive healthcare utilization [12]. As a result, an estimated 5- 10% and around 25% of hospitalized patients have developed HAIs in developed and developing countries, respectively [13].

Despite infection prevention and control being the main agendas of the national or global health community, the third quarter report showed the implementation level of the IPC program at Debre Tabor Comprehensive Specialized Hospital (DTCSH) is low among the components of the Ethiopian hospital transformation guidelines and was identified as a problem by program stakeholders during the Evaluability Assessment (EA). Ultimately, it will enhance the implementation status by providing the intended information to relevant stakeholders who have already been identified during the EA. So these evaluation study findings will also be useful for policymakers, health facility managers, healthcare workers, and evaluators studying this program in the future. In this evaluation, the identified stakeholders followed the nominal group technique in defining the problem, formulating evaluation questions, setting indicators for evaluation, and assigning weights to indicators and judgment parameters for evaluation. The identified stakeholders are presented in Table 1.

**Table 1:** Stakeholders analysis matrix for the ipc program at debre tabor comprehensive specialized hospital, Northwest ethiopia, 2022

Stakeholder	Role in the program	Interest or perspective on evaluation	Role in the evaluation	Communication strategy	Level of importance( H, M, L)
Amhara Regional Health Bureau	budget, supplies, training, supportive supervision, Planning and evaluation	To know the implementation status, best practices, and gaps in program implementation Use of findings for program improvement and decision making	Facilitate the evaluation process Identify and weigh indicators. Set judgement parameters	A formal letter Email through reading guidelines, policies	High
South Gondar Zonal Health Department	Supporter	Use of findings for program improvement and decision making	Face-to-face	Face-to-face presentation Email	High
Debre Tabor Comprehensive Specialized Hospital	Implementer	Use of findings for program improvement and decision making To identify strengths and weaknesses of the program implementation	Data source Formulating evaluation questions Identify weighting indicators Set judgment parameters	Formal letter Face-to-face presentation	High
Debre Tabor University	Supplies and training	Use of finding for program improvement and decision making	Facilitate evaluation process Formulating evaluation questions identify and assign a weight for indicators and set judgment parameters	Mail Face-to-face presentation	High
Debre Tabor Town Health Office	Supporter	To identify weaknesses and strength of the program implementation	Facilitate the evaluation process	Face-to-face presentation	Medium
Hospital Health Care Workers	Service delivery	To identify a skill gap To identify the strengths and weaknesses of the program implementation	Data Source Identify indicators Assign weight to indicators and set judgment parameters	Key informant interview Observation session/discussion	High
Clients and attendants	Service user	To get quality services	Source of information Formulating evaluation questions	Interviewer-administered interview	High

## Methods and materials

### Evaluation settings and period

This evaluation was conducted at Debre Tabor comprehensive specialized hospital, which is found in South Gondar Zone, Amhara national regional state, Ethiopia. It was established in 1930- 31 by Dr. Ogambic, a Norwegian missionary. The hospital has been providing services for a population of 2,651,350.

It served as a general hospital till the end of 2019 and upgraded to a comprehensive specialized hospital in 2020. It is also 97 km from Bahir Dar (the capital city of Amhara’s national regional state) and 666 km northwest of Addis Ababa (the capital city of Ethiopia).

The hospital is organized into wards for medical, surgical, pediatric, gynaecology, emergency, ophthalmology, and intensive care unit. It has a total of 125 inpatient beds in all wards. It has also hired a total of 534 people. Of these, 99 are administration staff; 16 specialists; 38 general medical practitioners; 05 emergency trained professionals; 11 health officers; 163 nurses; 38 midwives; 25 laboratory technicians and technologists; 31 pharmacists and pharmacy technicians; 08 x-ray professionals; 08 anaesthetists; 02 biomedical engineers; 08 HIT; 01 physiotherapist; 05 psychiatry; 05 optometry; 02 environmental health; 60 cleaners; and 17 other technical staff.

An Evaluability Assessment (EA) was conducted from October 15–30, 2021. Moreover, the evaluation was conducted from May 25 to July 5, 2022.

### Evaluation approach

A formative evaluation approach was employed to evaluate the implementation fidelity of IPPs at Debre Tabor Comprehensive Specialized Hospital. A formative evaluation was used to understand what was done and what was not done, and to identify barriers and enablers to IPP for the implementation of the program. Since the IPC program was in the implementation stage, the formative evaluation was particularly important for this evaluation [14].

### Evaluation design

A facility-based cross-sectional design with concurrent mixed methods was used to evaluate the implementation fidelity of the IPC program. A cross-sectional design was used to evaluate the implementation of IPPs at Debre Tabor Comprehensive Specialized Hospital. A cross-sectional design is composed of both qualitative and quantitative research methods that help to examine program implementation and answer the questions “why” and “how.” The aim of combining the qualitative method was to strengthen the credibility of the evaluation findings and answer the evaluation questions. A concurrent mixed method was employed to triangulate data between qualitative and quantitative findings [15].

### The focus of evaluation and dimensions

This evaluation focused on the process of the IPC Program, mainly the implementation fidelity of infection prevention practices and why and how the program was implemented in line with the national IPC guideline at DTCSH. To evaluate the implementation of the IPC program, the three constructs of adherence, participant responsiveness, and the facilitation strategy dimension of implementation fidelity were used [16–19].

### Populations and samplings

All clients who visit Debre Tabor specialized hospital; all healthcare providers and cleaners in Debre Tabor specialized hospital; the IPC focal person; all quality officers in the hospital; the matron; the hospital's medical director; and a hospital manager. The sample size for measuring the participant responsiveness dimension of the IPC program was calculated using a single population proportion formula. A 5% sampling error or precision at a 95% confidence interval and assuming that 50% of clients were satisfied with infection prevention practice are used in computing the sample size to achieve adequate precision [20].

The study's sample size was adjusted to account for the study population. The sample size was determined and calculated as follows:

$$\begin{array}{l}n=\frac{\left(Z\alpha/2\right)^2{\ast}P\left(1-P\right)}{d^2}=\frac{\left(1.96\right)^2\left(0.5\right)\left(0.5\right)}{\left(0.05\right)^2}=\bf{384.16}\approx\bf{384}\\\;\;\;=384+\left(384\,{\ast}\,10\%\, \text{of non-respondents}\right)=\bf{423}\end{array}$$

Clients admitted over 24 h in all wards participated in the study. Sample sizes were calculated from the previous year’s similar month’s patient data flow (920), and the calculated sample size was 423. The k^th^ interval $\approx 2$. The first case was selected using a simple random sampling technique by lottery, and the subsequent cases were selected every two case intervals using systematic sampling techniques using liaison inpatient admission registration.

A total of 35 non-participatory observation sessions were observed. Eleven key informants (one KII with a hospital manager, one KII with a medical director, one KII with a hospital matron, one KII with the IPC focal person, one KII with a hospital quality unit team leader, five KII with ward coordinators, and one KII with cleaning staff coordinators) were interviewed. Resource inventories were conducted in all wards, rooms, and hospital procurement models using an adapted checklist. Twelve months’ worth of documents such as the IPC Committee meeting agenda, IPC training registration, hospital quarterly review meeting agenda, hospital health education registration, and reports, were to examine adherence and facilitation strategies from the period of July 1, 2021, to June 30, 2022. Assigned hospital managers, medical directors, matrons, ward coordinators, and cleaning staff coordinators who were working for six months and above were included in the study. Clients who were admitted to the hospital for more than 24 h during the data collection period were also included in the study. However, clients who lost consciousness and clients whose age was under 18 years were excluded from the study.

### Data collection tools and procedures

A resource inventory assessment checklist was adopted to examine the infrastructure, resources, and equipment of the IPC program by referring to WHO infection prevention and control tools and national IPC guidelines [4, 21].

A data extraction checklist was used to extract data from documents (the IPC Committee agenda, the IPC training registration agenda, the hospital quarterly performance review agenda, the hospital health education registration, and the IPC supply procurement models) and was adopted by referring to the National IPC guidelines [22]. A structured observation checklist was prepared to assess the adherence of healthcare providers’ infection prevention practices to guidelines by referring to literature and the CDC Infection Prevention and Control Assessment Tool [23, 24]. A structured interviewer-administered questionnaire to national guidelines includes the following: socio-demographic characteristics of clients, infection prevention practices of clients, and satisfaction [25, 26]. A semi-structured key informant interview guide was prepared and contains the following: the backgrounds of the interviewee; information related to program management; strategies used to facilitate the IPC program; IPC training, activities, and training materials; IPC monitoring, feedback, and evaluation; and barriers and enablers to implementing the IPC program. A tool was prepared by referring to different guidelines and national IPC guidelines [21, 27].

### Data quality assurance

For quantitative data, a pre-test was done among 21 clients in the Debre Tabor Health Center to check the quality of the data collection tools. And then a correction was made based on the pre-test findings. The data collection tool contained the clients’ responsiveness dimension of implementation fidelity. Data collectors were supervised daily, and the questionnaires were checked for consistency and completeness of data after being sent from the data collectors to the principal evaluator.

### Data management and analysis

Completed data were checked for completeness and consistency, and it was cleaned. Then the quantitative data was downloaded from Kobo Tool Box software and imported into SPSS version 25 for analysis. For qualitative data, field notes and audio recordings were taken and transcribed every night before being analyzed using open code software version 4.02 software. Data cleaning was done by the principal evaluator after it was sent from the data collectors. For quantitative data, descriptive statistics (univariate analysis) were used to determine the frequency, mean, and proportion of variables. A binary logistic model was fitted. Variables having a p-value of less than 0.25 were candidates for multivariate analysis. In multivariable analysis, a p-value of less than 0.05 with a 95% confidence interval was taken as a factor that acts as a barrier and an enabler of participants’ responsiveness to the program. Participants’ responsiveness (satisfaction) was measured using a Likert scale (1 = very poor, 2 = poor, 3 = good, 4 = very good, and 5 = excellent) of alternatives. Each satisfaction from the 5-point Likert scale of total items was categorized into two (below the cut-off point dissatisfied and above the cut-off satisfied) using the demarcation threshold formula [(total highest score- total lowest score)/2] + total lowest score. Each satisfaction item was analyzed for frequency, mean, and standard deviation.

Qualitative data were transcribed, coded, thematized, and categorized using open-source software.

### Operational definitions

Adherence was used to measure the compliance of healthcare providers to infection prevention and control guidelines through the inventory checklist and non-participatory observation. We used 14 indicators to assess this, with a 30% weighting.

The strategy the hospital followed to facilitate IPC implementation was IPC training, monitoring, and feedback delivered to cleaning staff and healthcare providers to enhance the implementation of the IPC. To measure this, we used 10 indicators and gave them a weight of 25% by reviewing 48 documents.

Participant responsiveness of clients was measured by both practices of infection prevention and satisfaction with the cleanliness of the hospital using 12 indicators and had a weighted value of 35%. Satisfaction assessed how clients react to the infection prevention practices and their satisfaction was measured using a five-point Likert scale (1 = very poor, 2 = poor, 3 = good, 4 = very good, and 5 = excellent).

Participant responsiveness (client satisfaction) was the outcome variable. Clients’ overall satisfaction was dichotomized into satisfied and dissatisfied depending on the threshold, which was computed using the demarcation threshold formula: $\left[\frac{total\ highest\ score-total\ lowest\ score}{2}\right]+total\ lowest\ score$.

The independent variables of participant responsiveness were socio-demographic variables (age, sex, educational level, marital status, and family income) and the availability of hand-washing facilities.

The overall implementation fidelity of infection prevention practice was measured using 36 indicators over the three dimensions (adherence, participant responsiveness, and facilitation strategy) of fidelity. The status of infection prevention implementation was categorized and rated below 50 as low, 50–75 as medium, and ≥ 75 as high by program stakeholders.

Indicators were adapted from national and WHO IPC guidelines, and for each indicator, a score was calculated using the formula: $indicator\;score=\frac{Observed\; number\times }{indicator\;weight}$ 100.

### Judgment matrix of analysis

The judgment parameters were determined based on the calculated indicator score adapted from measuring the implementation fidelity of the student affairs program [28]. The adherence, participant responsiveness, facilitation strategy dimensions, and overall program fidelity were classified as low (less than 50%), medium (50–74.9%), and high (greater than 74.9%). Based on the stakeholders’ agreement, the weighted value given for adherence was 40%, participant responsiveness was 35%, and facilitation strategy was 25%.

### Evaluation dissemination plan

The evaluation findings were reported and submitted to the University of Gondar, the College of Medicine and Health Sciences, and the Department of Health Systems and Policy. Then the approved evaluation findings were disseminated to the Amhara regional health bureau, Debre Tabor comprehensive specialized hospital, South Gondar Zonal Health Department, and other stakeholders through different means of communication like face-to-face, hard copy presentation, and publication.

## Results

In total, 423 clients who admitted to having waited more than 24 h were interviewed, with a 100% response rate. Thirty-five non-participatory observations were also conducted in five wards. 11 key informants were interviewed, and resource inventory and document review (from July 1, 2021, up to June 30, 2022) were conducted.

### Adherence to the IPC programme

According to the results of the inventory checklist, the hospital had an IP committee, an IPC team, and an IPC focal person with defined and approved roles and responsibilities, but the IP committee did not actively support an IPC team.

This was supported by the key informant interview result: there is an IPC committee established from different professions based on the terms of reference of the IPC guidelines, but the committee does not meet periodically.

“*The IPC Committee meeting was interrupted due to some interference, but there are rules or terms of reference set on the IPC guidelines. The meeting should be conducted every month, and it is difficult to say that it has been in the IPC Committee before, but it will be strengthened in the future”.*

[32-year-old male Public Health Officer on the Quality Team].

The facility leadership did not support the IPC program by allocating an adequate budget. The hospital has IPC guidelines consistent with national guidelines that include both transmission-based precautions and standard precautions under them.

Due to a budget deficit, the three key informants interviewed stated that the hospital administration has not allocated an adequate budget for the IPC program.

*“Already, we have established an IPC Committee in the hospital. The committee plans and acts in accordance with its plans; however, due to budget constraints and other factors, we are not adequately supporting the IPC program".*

[38 years male environmental health professional].

The water service in the hospital is continuously available at least five days per week at all times, and a sufficient power supply is available for pumping, boiling water, and sterilization of medical devices and equipment.

The hospital rooms were built with natural ventilators, but they had no mechanical ventilators except in pharmacy store rooms. The hospital also has different isolated rooms for cohorting patients with similar pathogens. Cleaning materials, e.g., detergents, mops, and buckets, are readily available for the cleaning staff, and personal protective equipment is also sufficiently available for the cleaning staff at all times.

This is congruent with the key informant's findings that "if there is not an adequate supply of inputs and materials, we will be the first victims, and patients and professionals will be harmed next to us." So we avail cleaning materials and inputs sufficiently". *[30 year-old male cleaning staff coordinators]*.

**Fig. 2:**
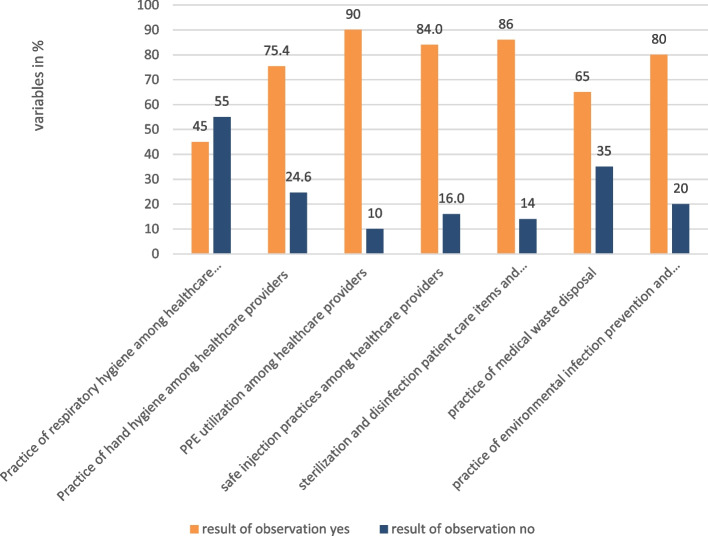
Observations on the practice of Infection Prevention at DTCSH, 2022

Healthcare providers adhered to the guidelines by implementing 45 percent of respiratory hygiene, 75.4 percent of hand hygiene practice, 90 percent of PPE utilization, 84 percent of safe injection practice, and 86 percent of sterilization and disinfection of patient care devices and equipment. On the other hand, cleaners adhered to the guideline in environmental infection prevention and control practice by 80 percent (Fig. 2). A summary of adherence indicators is presented in Table 2.

**Table 2:** Summary of adherence dimension indicators at DTCSH, 2022

S.No	Indicators	E^a^	O^a^	W^a^	S^a^	A^a^	JP^a^
1	The proportion of functional hand hygiene facilities in the hospital	13	12	3	2.77	92	High
2	% of toilets with functional hand hygiene facilities in the hospital	15	13	3	2.6	86.67	High
3	% of HCPs adhered to respiratory source control measures	5	3	2.5	1.5	60	High
4	% of HCPs who offered masks to coughing patients	5	2	2.5	1	33	Low
5	% of HCPs wash their hands before contacting w patient	5	2	2.5	1	33	Low
6	% of HCPs washed their hands after contacting a patient	5	4	2.5	2	80	high
7	% of HCPs who wore a mask during the interview	5	4	2.5	2	80	High
8	% of HCPs prepare injections using an aseptic technique	5	5	3	3	100	High
9	% of HCPs disposed of all sharps in a safety box immediately after injection	5	3	3	1.8	60	Medium
10	% of HCPs adhered to a single-patient use	5	5	2.5	2.5	100	High
11	The proportion of HCPs injections administered	5	3	3	1.8	60	Medium
12	The Proportion of HCPs adhering to work practice control measures	5	2	3	1.2	40	Low
13	The proportion of HCPs used the three-bin system to segregate wastes	5	3	4	2.4	60	Medium
14	Proportion of cleaners engaged in environmental cleaning by wearing appropriate PPE	5	5	3	3	100	High
	Overall			40	28.57	71.43	Medium

E^a^ Expected, O^a^ Observed, W^a^ Weight, S^a^ Score, A^a^ Achievement in percentage(S/W*100), JP^a^, Judgment Parameter

### Participant responsiveness of the IPC Program

#### Socio-demographic characteristics of respondents

Table 3 shows the socio-demographic characteristics of clients who participated in the responsiveness dimension of fidelity. More than half of the respondents (54.61%) were admitted to both pediatric and medical wards almost equally, whereas 2.6% of clients were admitted to the intensive care unit of the pediatric ward. Around thirty percent of study participants were unable to read and write, and 9.93 percent could read and write.

**Table 3:** Socio-demographic characteristics of clients at DTCSH, 2022

Variable		Frequency (*N* = 423)	Percentage
Ward admitted	Pediatric	116	27.42
	Medical	115	27.19
	Surgical	87	20.57
	Gynaecology-Obstetric	64	15.13
	Orthopaedic	30	7.09
	Intensive Care Unit (ICU)	11	2.6
Educational level	Unable to read and write	127	30.02
	Primary Education	119	28.13
	College and above	90	21.28
	Secondary Education	42	9.93
	Read and write	45	10.64
Age	≤ 34	238	56.26
	> 34	185	43.74
Sex	Female	226	53.43
	Male	197	46.57
Residence	Rural	216	51.06
	Urban	207	48.94
Marital status	Married	339	80.14
	Single	67	15.84
	Separated/Divorced	12	2.84
	Widowed	5	1.18
Religion	Orthodox	416	98.35
	Muslim	6	1.42
	Protestant	1	0.24
Occupational Status	Farmer	208	49.17
	Government employee	69	16.31
	Housewife	47	11.11
	Merchant	32	7.57
	Daily labourer	15	3.55
	Other^a^	52	12.29
Family monthly income in ETB	Less than 1000 ETB	17	4.02
	1000–2500 ETB	169	39.95
	2501–5000 ETB	145	34.28
	Greater than 5000 ETB	92	21.75

^a^Other includes self-employed, students, private workers and drivers

The age of the respondents ranged between 18 and 80, and the mean age was 34.44. Nearly 55% of them were female, and 51.06% lived in rural areas.

Of a total of 423 respondents, 80.14 percent were married, and the majority of them (98.35%) were orthodox. Regarding family monthly income, 40% and 4% of study participants earned in the range of 1000 and 2500 Ethiopian birr and less than 1000 Ethiopian birr per month, respectively.

#### Clients’ infection prevention practice

Of the clients interviewed, around 22 percent got information on how to prevent HAIs from healthcare providers by using face-to-face health education methods (98.91 percent) and by using materials such as flip charts, brochures, and leaflets prepared in the Amharic language (1.09 percent). The majority (82.98 percent) did not also wear a mask or mouth and nose cover due to unavailability (43.30 percent), uselessness (29.63 percent), inconvenience (14.25 percent) and other (12.82 percent) reasons.

Around thirty percent of respondents did not wash their hands with soap and water due to the unavailability of soap. Whereas, nearly 5% and 16% of clients used hand sanitizer and put waste separately in prepared waste bins, respectively (Table 4).

**Table 4:** Clients’ infection prevention practices at DTCSH, 2022

Items	Frequency	Percent (%)
**Do you get information on how to prevent HAIs from healthcare providers?**		
Yes	92	21.75
No	331	78.25
**If ‘yes’ to the above question which educational materials are used?**		
Face-to-face health education	91	98.91
Using materials prepared in Amharic language (flip chart, brochure,)	1	1.09
**Do you wear a mask/ a mouth and nose cover?**		
Yes	72	17.02
No	351	82.98
**If ‘no’ to the above question what are the reasons for not wearing the mask**		
Unavailability	152	43.30
Uselessness	104	29.63
Inconvenience	50	14.25
Other*	45	12.82
**Do you wash your hands with soap and water between activities?**		
Yes	297	70.21
No	126	29.79
**What are the reasons not for not applying the soap?**		
Unavailability of soap	126	100
**Do you prevent yourself from injury with sharp instruments?**		
Yes	319	75.41
No	104	24.59
**If ‘yes’ how do you protect yourself from injury**		
By avoiding touching sharp instruments	296	69.98
By wearing gloves	23	5.44
**If ‘no’ what are the reasons**		
Lack of protective equipment	90	86.54
They have no risk	14	13.46
**Do you use hand sanitizer to reduce your risk of infection?**		
Yes	19	4.49
No	404	95.51
**If ‘no’ what are the reasons not to use hand sanitizer**		
Unavailability	317	78.47
Inconvenience	76	18.81
Other^a^	11	2.72
**Do you put wastes separately in different waste bins?**		
Yes	69	16.31
No	354	83.69
**If ‘no’ what are the reasons to put waste separately**		
Lack of awareness	343	81.09
Lack waste container	11	2.6

Other^a^ = lack of awareness, negligence, religion and due to emergency, no importance, lack of awareness and due to emergency

Among the total participants, nearly 5% and 77% of clients responded excellently and very good to the cleanliness of the hospital surroundings, respectively. Accordingly, less than 5% of clients felt discomfort, with a mean of 3.82 and an SD of 0.588.

Regarding the cleanliness of the toilet in the hospital, only 1.65% of respondents said it was excellent, but 15.37% of them said it was very poor, with a mean of 2.67 and SD of 1.135 (Table 5).

**Table 5:** Level of client satisfaction with the cleanliness of the hospital, June 2022

Satisfaction item	Excellent (%)	Very good (%)	Good (%)	Poor (%)	Very poor (%)	Mean	SD
How do you feel about the cleanliness of the hospital surrounding	21(4.96)	326(77.07)	57 (13.48)	18(4.26)	1(0.24)	3.82	± 0.588
How satisfied are you with the hospital premises area?	20(4.73)	312(73.76)	73 (17.26)	17(4.02)	1(0.24)	3.79	± 0.598
How satisfied are you with the cleanliness of waiting room area?	21(4.96)	259(61.23)	119 (28.13)	23(5.44)	1 (0.24)	3.65	± 0.671
How satisfied are you with the cleanliness of the corridors area?	19(4.49)	258(60.99)	127 (30.02)	18(4.26)	1(0.24)	3.65	± 0.646
How satisfied are you with the cleanliness of the toilets?	10(2.36)	105(24.82)	128 (30.26)	95(22.46)	85(20.09)	2.67	± 1.135
How satisfied are you with the cleanliness of hospitals?	11(2.6)	175(41.37)	207 (48.94)	26(6.15)	4(0.95)	3.39	± 0.685
How satisfied are you with cleanliness of beds?	10(2.36)	198(46.81)	196 (46.34)	18(4.16)	1(0.24)	3.47	± 0.630
How satisfied are you with cleanliness of linen?	9(2.13)	169(39.95)	174 (41.13)	66(15.6)	5(1.18)	3.26	± 0.788
How satisfied are you with cleanliness of pyjamas?	7(1.65)	136(32.15)	208 (49.17)	66(15.6)	6(1.42)	3.17	± 0.757
How satisfied are you with cleanliness of the staff gowns?	14(3.31)	199(47.04)	201 (47.52)	8(1.89)	1(0.24)	3.51	± 0.607
How satisfied are you with hygiene of the staff?	15(3.55)	211(49.88)	188 (44.44)	8(1.89)	1(0.24)	3.55	± 0.610
How satisfied are you with cleanliness of materials availed by the hospital?	12(2.84)	192(45.39)	206 (48.7)	13(3.07)	0	3.48	± 0.607
How do you satisfied with the food services availed by the hospital?	4(0.95)	159(37.59)	239 (56.5)	19(4.49)	2(0.47)	3.34	± 0.602
How satisfied are you with the information provided regarding waste segregation, norms of the ward and infection prevention by the staff?	2(0.47)	69(16.31)	275 (65.01)	77(18.2)	0	2.99	± 0.604

Clients’ overall satisfaction was dichotomized into satisfied and dissatisfied depending on the threshold, which was computed using the demarcation threshold formula: $\left[\frac{\boldsymbol{total}\;\boldsymbol{highest}\;\boldsymbol{score}-\boldsymbol{total}\;\boldsymbol{lowest}\;\boldsymbol{score}}{\textbf{2}}\right]+\boldsymbol{total}\;\boldsymbol{lowest}\;\boldsymbol{score}$ [29]. Clients’ overall satisfaction was presented in Table 6.

**Table 6:** Shows the clients’ satisfaction category for each satisfaction item on the IPC practiced at Debre Tabor comprehensive specialized hospital in 2022

S.No	Satisfaction item	Satisfaction category (*N* = 423)	
		Satisfied (%)	Dissatisfied (%)
1	How satisfied are you with the cleanliness of hospital surroundings?	404(95.51)	19(4.49)
2	How satisfied are you with the cleanliness of hospital premises?	405(95.74)	18(4.26)
3	How satisfied are you with the cleanliness of the waiting room area?	399(94.33)	24(5.67)
4	How satisfied are you with the cleanliness of the corridors?	404(95.51)	19(4.49)
5	How satisfied are you with the cleanliness of toilets?	243(57.45)	180(42.55)
6	How satisfied are you with the cleanliness of bathrooms?	393(92.91)	30(7.09)
7	How satisfied are you with the cleanliness of the beds?	404(95.51)	19(4.49)
8	How satisfied are you with the cleanliness of the linen?	352(83.22)	71(16.78)
9	How satisfied are you with the cleanliness of your pyjamas?	351(82.98)	72(17.02)
10	How pleased are you with the cleanliness of the staff gown?	414(97.87)	9(2.13)
11	How satisfied are you with hygiene of the staff?	414(97.87)	9(2.13)
12	How satisfied are you with the cleanliness of materials available at the hospital?	410(96.93)	13(3.07)
13	How satisfied are you with the cleanliness of the diet provided by the hospital?	402(95.04)	21(4.96)
14	How satisfied are you with the information provided regarding waste segregation, the norms of the ward, and infection prevention provided by the staff?	346(81.80)	77(18.20)

**Fig. 3:**
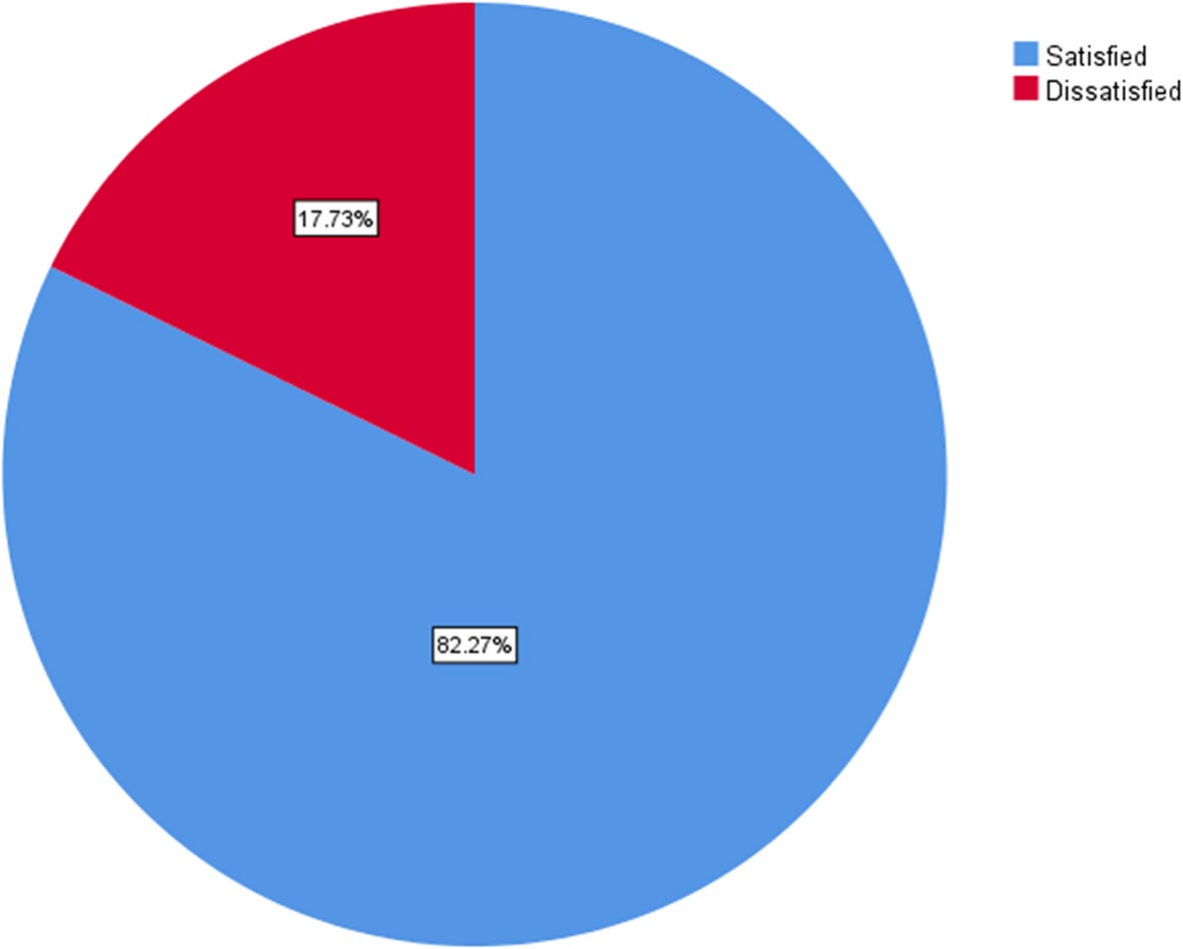
Clients’ overall satisfaction with infection prevention practice at DTCSH in 2022

Overall, 348 (82.3%) clients were satisfied with the infection prevention practices in the hospital (Fig. 3).

#### Factors associated with clients’ satisfaction with IPC practice

In binary logistic regression analysis, ward, educational status, residence, occupation, health education, and availability of water at the hospital have a p-value of less than 0.25 and are considered candidate variables for multivariable analysis.

In a multivariable analysis, ward admission and educational level (both with a p-value of 0.05) were statistically related to client satisfaction with infection prevention practices in the hospital. The odds of IPPs satisfaction among clients admitted to a medical ward were 0.36 compared with clients admitted to a surgical ward (AOR = 0.358, 95%CI: 0.165–0.777). The odds of IPPs satisfaction among clients admitted to the Gynecology-Obstetrics ward were also 0.32 compared with the IPPs satisfaction of clients admitted to a surgical ward (AOR = 0.315, 95% CI: 0.120–0.827). Moreover, the odds of IPPs satisfaction among clients admitted to the orthopedics ward were 0.12 compared with the odds of IPPs satisfaction among those admitted in a surgical ward (AOR = 0.117, 95%CI: 0.014- 0.940).

In terms of educational level, the odds of satisfaction with infection prevention practices were 3.5 when compared to the odds of satisfaction with IPPs among those who could not read or write (AOR = 3.522, 95%CI: 1.574- 12.991). And the odds of IPPs satisfaction among clients who attended college and above were 3.1 compared to the odds of IPPs satisfaction among clients unable to read and write (AOR = 3.071, 95%CI: 1.638–10.119) (See Table 7).

**Table 7:** Multivariable analysis result of clients’ satisfaction with IPC practice at DTCSH, 2022 (*n* = 423)

Variables	Satisfaction category		95% confidence interval		*P*-Value
	Satisfied (%)	Dissatisfied (%)	COR	AOR	
**Ward admitted**					
Surgical	65(15.4)	22(5.7)	1	1	
Medical	103(24.3)	12(2.8)	0.342(0.26–0.313)	0.358(0.165–0.777)	**0.009**
Intensive Care Unit(ICU)	6(1.4)	5(1.2)	2.167(0.40–4.778)	2.302(0.035–4.621)	0.277
Gyn-Obs	56(13.2)	8(1.9)	0.411(0.196–1.169)	0.315(0.120–0.827)	**0.019**
Pediatric	96(22.7)	20(4.7)	0.618(0.349–1.397)	0.549(0.262–1.1510	0.112
Orthopaedic	23(5.4)	7(1.7)	0.116(0.015–0.902)	0.117(0.014–0.940)	**0.044**
**Educational level**					
Unable to read and write	112(26.5)	15(3.5)	1	1	
Able to read and write	37(8.7)	8(1.9)	1.59(0.152–3.648)	0.821(0.162–4.157)	0.812
Primary education level	104(24.6)	15(3.5)	1.015(0.589–3.907)	1.592(0.601–4.217)	0.349
Secondary education	30(7)	12(2.8)	3.011(1.559–11.589)	3.522(1.574–12.991)	**0.005**
College and above	65(15.4)	25(5.9)	2.912(2.032–11.399)	3.071(1.638–10.119)	**0.003**
**Occupation**					
Government employee	52(12.3)	17(4)	1	1	
Farmer	189(44.7)	20(4.7)	0.332(0.150–0.709)	1.098(0.321, 3.760)	0.881
Merchant	27(6.4)	7(1.7)	0.778(0.222–2.067)	0.838(0.234, 2.999)	0.786
House wife	37(8.8)	8(1.9)	0.672(0.164–1.474)	0.754(0.214, 2.659)	0.661
Daily laborer	7(1.7)	7(1.7)	3.13(0.789, 301)	1.901(0.231, 3.21)	0.999
Other	36(8.5)	16(3.8)	1.310(0.555–3.092)	2.519(0.847, 7.493)	0.097
**Health education for clients**					
Yes	75(17.7)	17(4)	1	1	
No	273(64.6)	58(13.7)	0.943(0.854–4.500)	2.156(0.879–5.288)	0.093
**Water availability**					
Yes	331(78.3)	56(13.2)	1	1	
No	17(4)	19(4.5)	6.593(0.547–8.720)	2.462(1.048–6.373)	0.052

Other^a^ = : 1 = reference

*COR* Crude odds ratio and *AOR* Adjusted odds ratio

Accordingly, the overall measurement of the participant responsiveness dimension was 60.6%, which was judged as a medium using 12 indicators (Table 8).

**Table 8:** Summary of participant responsiveness indicators in DTCSH, 2022

**S.No**	Indicators	E^a^	O^a^	W^a^	S^a^	A^a^	JP^a^
1	% of patients received education on how to prevent HAIs from healthcare providers	423	92	4	0.9	22.5	Low
2	% of patients who wore a mask during the interview	423	72	2.5	0.4	16	Low
3	% of patients who wash their hands with soap and water in the hospital	423	297	3.5	2.5	71.4	Medium
4	% of patients who avoid touching anything without wearing a glove	423	123	2	0.6	30	Low
5	% of patients who apply sanitizer to reduce the risk of infection	423	19	1.5	0.1	6.7	Low
6	% of patients who discard waste in a black coloured waste container	423	63	2.5	0.4	16	Low
7	% of patients satisfied with the cleanness of the toilet	423	243	4	2.3	57.5	Medium
8	% of patients satisfied with the cleanness of the hospital compound	423	404	3	2.9	96.7	High
9	% of patients satisfied with the cleanness of the hospital rooms	423	399	3	2.8	93.3	High
10	% of patients satisfied with the cleanness of the hospital beds	423	404	3	2.9	96.7	High
11	% of patients satisfied with food hygiene served in the hospital	423	402	3	2.9	96.7	High
12	% of patients satisfied with hospital bed linen of the hospital	423	352	3	2.5	83.3	High
	Overall participant responsiveness			35	21.2	60.6	Medium

E^a^ Expected, O^a^ Observed, W^a^ Weight, S^a^ Score, A^a^ Achievement in percentage(S/W*100), JP^a^ Judgment parameter

### Strategies and Factors of the IPC Program

#### Registration and report review

In the study, a total of 100 cleaners were employed in the hospital, but 60 cleaners were found at work at the time of the study because the number of cleaners had reduced from 100 to 60. All 100 cleaning staff received the first round of IPC training in a year, and 100% of staff followed the second round of training in a year. According to a key informant interview, all the cleaning staff received IPC training every six months.

*“When we joined the hospital for the first time, IPC training was given to us initially. Then, every six months, we all go through IPC training. [30-year-old male, grade 10-cleaning staff].*

Of 375 healthcare providers, 80 (21.33%) and 10 (2.67%) were trained on IPC for the first time and for the second time in a year, from August 1, 2021, to June 30, 2022, in the hospital, respectively. The majority of the healthcare workers have not received the training, as indicated by most key informants. “M*ost of us did not get IPC training, including me. It is better to say no trained healthcare providers”. [A 29-year-old male BSc Nurse Professional].*

According to the IPC committee meeting agenda review, there was a schedule to conduct IPC meetings every month by the IPC committee. From the twelve times the committee expected to meet in a year, they met four times (33.33%). The agenda for the hospital's quarterly review meeting was reviewed, and the hospital's performance should be evaluated every quarter rather than four times (75% of the time) in a year.

As reported by all interviewees, the IPC program was monitored and evaluated separately by the IPC Committee and with other activities every month and every quarter, respectively.

“*First, an IPC performance plan will be prepared, and then the activities will be monitored and evaluated in accordance with the plan by the IPC Committee during its monthly meeting. And it will be evaluated also jointly with other activities, but we are not meeting based on the schedule”.*

*[35 years male general physician]*

Forty-Eight sessions of health education should be delivered to clients about IPC from August 1, 2021, to June 30, 2022. It was given four times to 240 males and 180 females, for a total of 420 clients and visitors, based on the review of the health education package in the hospital and hospital annual report. In the past year, no hand hygiene assessment was done on healthcare providers.

Of the five wards, four had IPC guidelines in their working departments, and three wards posted hand hygiene promotion posters in their hand hygiene stations (Table 9).

**Table 9:** Document review on facilitation strategies of the IPC program in DTCSH, June 2022

Activities reviewed/checked on documents	Required in a year	Achieved in a year
The number of cleaning staff trained on IPC this year	100	100
The number of cleaning staff trained on IPC two times in this year	100	60
The number of HCPs trained on IPC this year	375	80
The number of HCPs trained on IPC two times in this year	375	10
The number of monthly meetings conducted by IPC Committee	12	4
The number of performance evaluations conducted by the hospital this year	4	3
The number of health education sessions about IPC given to clients this year	48	4
The number of hand hygiene assessments conducted by the hospital in this year	4	0
The number of wards with IPC guidelines	5	4
The number of wards posted Hand hygiene poster in the hand hygiene station	5	3

Eleven key informants—the hospital manager, medical director, matron, quality team leader, IPC focal person, coordinators of the medical ward, surgical ward, gynecology-obstetric ward, orthopaedic ward, paediatric ward, and cleaning staff responded to the interview, and no participant disagreed to participate in the study. Among the participants, all of them were male, and only one respondent was in grade 10, and the other had a diploma or higher in educational status (Table 10).

**Table 10:** Background of key informant interviewees at DTCSH, June 2022

Profession	Sex	Educational level	Number of participants	IPC training status				Ave. service years
				Untrained	Only Onsite	Only Offsite	both	
**Nurse**	Male	Diploma = 01	06	04	02	0	0	2.42 years
		1^st^ degree = 05						
**Environmental Health**	Male	2^nd^ degree	02	0	0	0	02	2.25 years
**Doctor**	Male	1^st^ degree	01	0	0	0	01	2 years
**Health Officer**	Male	2^nd^ degree	01	0	01	0	0	4 years
**Cleaner**	Male	Grade 10	01	0	01	0	0	2 years
**Total**			11	04	04	0	03	

#### Barriers and enablers of infection prevention and control practices

The major themes that emerged in the qualitative data analysis were healthcare worker-related factors, institutionally-related factors, management-related factors, and patient- and visitor-related factors.

#### Healthcare workers’ related factor

As described by some participants, professionals’ awareness of infection prevention and control was poor, and they had poor utilization of provided resources. But there are no factors mentioned as enabling factors on the healthcare providers’ side.

*“The healthcare providers’ awareness of IPC practice was poor.”* (*26-year-old male, environmental health*). *“The problem with health care providers’ attitude is huge.” (32-year-old male, health officer). “There is a problem with professional IPC supply utilization as an owner” (38-year-old male, environmental health).*

#### Institutional related factors

The unavailability of facilities such as insufficient showers and hand hygiene stations and some old buildings that are not comfortable to apply the current IPC hospital standards were the barriers mentioned as an obstacle to implementing infection prevention by almost half of the interview participants.

On the other hand, the hospital’s trend towards better achievement in the national hospital competition was responded to as a good opportunity to practice infection prevention standards.

*“Our hospital is in competition with the national hospitals, and this is a good opportunity for the health care providers to use this experience as a trend”.*

*[35-year-old male, general physician]*

#### Management-related factors

Most key informants described poor traffic flow management, inadequate provision of IPC supply and equipment, and a budget deficit for the program as the barriers to implementing the required standards. However, the management and matron were receptive to feedback.

*“From hospital management to ward coordinators, answers to questions are enthusiastic.”*

*[38-year-old male, BSc nurse professional].*

#### Patient- and visitor-related factors

As pointed out by some participants, the patient flow in the hospital was high and was a barrier to IPC practice.

*“There is a very high caseload of patients. It is a little difficult to give health education about IPC to every patient that comes because there is an incompatibility between case flow and the hospital staff. Healthcare providers became tired of delivering education to all incoming patients”. *

*[28 years old male Midwifery professional].*

The barriers and facilitators of IPC were presented in Table 11.

**Table 11:** Barriers and facilitators of infection prevention practice at DTHSC, June 2022

	**Healthcare worker-related factors**	**Institutional- related factors**	**Management- related factors**	**Patient-and visitor-related factors**
**Barriers**	Negative attitude towards IPC practice Poor utilization practice Lack of motivation The number of healthcare providers and cleaners was low	Insufficient shower and hand hygiene stations, an old building, Shortage of inputs	Budget problem, Poor traffic management	high patient flow,
**Facilitators**	The presence of competition	The hospital trends in national hospital competition	Management is receptive to feedback	

The hospital’s overall facilitation strategy achievement was 48%, which is low according to the decision parameter (Table 12).

**Table 12:** Performance of facilitation strategies’ indicators in DTCSH, June 2022

S.No	Measuring indicators	Expected in #	Observed in #	Weight (W)	Score(S)	Ach. In percentage (S/W $$*100)$$	Judgment parameter
1	% of cleaners trained on IPC training this year	100	100	2	2	100	High
2	% of cleaners trained on IPC two times this year	100	60	2	1.2	60	Medium
3	% of HCPs trained on IPC this year	375	80	1.5	0.3	21	Low
4	% of HCPs trained on IPC two times this year	375	10	1.5	0.1	7	Low
5	% of monthly meetings done by IPC Committee in the year	12	4	3	1	33	Low
6	% of performance evaluations conducted by the hospital this year	4	3	3	2.25	75	Medium
7	% of health education delivered about IPC to clients this year	48	4	3	0.25	8.3	Low
8	% of hand hygiene Assessments conducted by the hospital in this year	4	0	2	0	0	Low
9	% of wards had IPC guidelines in their working areas	5	4	3.5	2.8	80	High
10	% of wards posted the IPC hand hygiene posters in washing stations	5	3	3.5	2.1	60	Medium
	Overall			25	12	48	Low

The overall implementation fidelity of the IPC programme is 61.8 percent, which, based on the judgement parameters, is medium and needs improvement (Table 13).

**Table 13:** Summary of overall performance implementation indicators of process of the IPC program in DTCSH, June 2022

S.No	Dimensions	Relative weight (W)	Score(S)	Achv.in % (S/W*100)	Judgment parameter
1	Adherence	40	28.57	71.43	Medium
2	Participant responsiveness	35	21.2	60.6	Medium
3	Facilitation Strategies	25	12	48	Low
	Overall Process of IPC Implementation	100	61.77	61.77	50–74.9 = needs improvement

## Discussion

The implementation fidelity of the IPC program was judged using the three dimensions of adherence, participant responsiveness, and facilitation strategies. In this evaluation, the overall implementation of the IPC program was 61.77% using predetermined judgment parameters as set by stakeholders. As a result, it was rated as "medium, indicating that the program requires improvement. The level of adherence to national IPC guidelines by healthcare providers was 71.43%, which was deemed medium. Client responsiveness to IPC program implementation was 60.6%, and the hospital's strategy to facilitate IPC program facilitation was 48%, these were rated as medium and low on the judgement parameter, respectively.

The hospital’s adherence to the recommended IPC guideline was 81.8%. The hospital had an IPC committee, an IPC team, and an IPC focal person with defined and approved roles and responsibilities, but the IPC committee did not actively support the IPC team, and the facility leadership did not support the IPC program by allocating an adequate budget. This is due to an insufficient budget allocation by the Amhara regional health bureau and the lack of attention given by the government.

Furthermore, the hospital had an IPC guideline, a continuous water supply (for hand washing, drinking, and taking baths), and a sufficient power supply (for pumping, boiling water, and sterilization of medical devices and equipment). These findings were congruent with the assessment of IPC implementation in primary and secondary healthcare facilities in Nigeria: 80.8% of healthcare facilities had running water and soap in sufficient quantity, and 94.9% of healthcare facilities had hand hygiene stations at strategic places [30]. One possible explanation for this similarity is that the IPC program gained traction in both countries.

Healthcare providers adhered to IPC guidelines by applying 45% of respiratory hygiene, 75.4% of hand hygiene standards, 90% of the necessary PPE utilization, 86% of safe injection practice, and 65% of disinfection and sterilization of patient care devices and equipment in the hospital. In disposing of medical waste, more than half of the recommended activities and 80% of environmental infection prevention and control activities were completed in line with the guidelines. More than half of the recommended activities and 80% of environmental infection prevention and control activities were carried out in accordance with the guidelines when disposing of medical waste [4, 22]. Compared to the study conducted in Iraq, the practice of hand hygiene, PPE utilization, and medical waste disposal were lower in this hospital, but the practice of safe injection was higher (the practice of hand hygiene was 74.33%, PPE utilization was 77.33%, the practice of aseptic technique was 84%, and the practice of medical waste disposal was 96.33%) [23]. The reason for this variation might be differences in the economies of Iraq and Ethiopia.

Generally, clients’ infection prevention practices were low. 78.3% of clients did not have information on how to prevent healthcare-acquired infection; around 83 percent of clients did not wear a mask or mouth and nose cover; nearly 96 percent of clients did not use hand sanitizer to reduce infection; 84 percent of clients did not know how to put wastes separately using coded waste bins; and about 30 percent of clients did not wash their hands with soap and water. The national IPC guideline, on the other hand, recommended four components to the client empowerment process: patient understanding of their own role; client acquisition of sufficient knowledge in their ability to involve their healthcare providers; clients' knowledge and skills; and the presence of a facilitating environment [22].

Concerning the satisfaction of clients, overall hospital cleanliness was high, accounting for around 82 percent. And areas with the highest satisfaction gained from clients were the cleanliness of staff gowns and the hygiene of staff, both of which scored around 98 percent, and the dietary hygiene provided by the hospital, which scored around 97 percent. Toilet cleanliness was the least satisfactory area, scoring around 57 percent out of all satisfaction questions. The finding of our study was similar to that of a study done on patients’ perceived satisfaction with cleanliness in a tertiary care hospital in Udupi district, Karnataka state, India, which showed 99% of participants reported that the staff’s hygiene was clean and 46 percent of participants reported that the toilet needed more cleaning [26]. This similarity might be related to clients’ perceptions of congruency, even if the healthcare setup and services are not the same. Based on these evaluation findings, ward admission and educational status were statistically significant factors in the client’s satisfaction with the implementation of the IPC program in Debre Tabor Comprehensive Specialized Hospital.

Clients admitted to a surgical ward were less likely to be satisfied with infection prevention practices at the hospital than clients admitted to a medical, gynaecology-obstetric, or orthopaedic ward. This might be the IPC program well implemented in a medical, Gynecology-obstetric, and orthopaedic ward compared with a surgical ward.

The satisfaction with infection prevention practices among clients who attended secondary school and college or above was higher compared with those who could not read and write. The possible reason behind this might be that being educated increases the ability to compare the services delivered at different setups.

The evaluation findings indicated that IPC training was delivered twice a year to cleaning staff by the hospital. All cleaners (100%) participated in a training organized for the first round, but only 60% of them were trained in the second round. This is because the cleaning staff number was reduced from 100 to 60 following budget inadequacy in the hospital. The findings were consistent with the national IPC guidelines. On the contrary, from a total of 375 healthcare providers, around 21 percent of them received training in the first round, and around 3 percent received training in the second round. This was minimal according to the national guidelines. These low-trained healthcare providers implied that the hospital’s fidelity to its support implementation strategy was not in accordance with IPC standards. Like the study done in Ghana, only 35.7% of facilities and healthcare workers received periodic training related to new or updated IPC [31].

In addition, the national IPC guideline recommends IPC committee meetings be conducted every month, but the hospital IPC committee conducts meetings four times a year. And the hospital was evaluated three times at the quarterly review meetings in a year. The first quarterly review meeting was not evaluated due to the war of internal national crises.

Infection prevention and control were prioritized as a topic of health education to teach to hospital clients and visitors per week. However, the report revealed that the topic is given to clients and visitors four times out of a scheduled 48 times a year. There was an IPC guideline in all wards except the gynaecology-obstetric ward. Similarly, hand hygiene posters were posted in three wards: medical, orthopaedic, and paediatric wards [4, 22].

At Debre Tabor Comprehensive Specialized Hospital in Ethiopia, qualitative data analysis showed that the barriers to IPC practice in the hospital were HCPs’ poor attitude towards IP, inappropriate IPC supply utilization by HCPs, and a low number of HCPs and cleaning staff. These were thematized under healthcare worker-related factors, institutional-related factors like an insufficient number of showers and hand hygiene stations, old buildings, and a shortage of inputs for IPC practice, management-related factors like poor traffic management and an inadequate budget, and patient- and visitor-related factors like high patient flow. These findings lend support to the step-by-step implementation of the IPC program. Facilitators identified include the presence of competition among wards, the hospital’s previous trend in national hospital competition awards, and the hospital management’s receptivity to forward feedback. These facilitators, unlike those studied in Jimma’s specialized hospital, had an adequate budget and constructed a new hospital building with improved, albeit inconsistent, water access and adequate environmental cleaning [32]. In a qualitative study at Amhara teaching hospitals in Ethiopia, the barriers and facilitators to IPC practice were also thematized as organizational, healthcare workers, patients, and visitors [32, 33].

### Limitations of the evaluation

The findings of this study were limited to Debre Tabor Comprehensive Specialized Hospital. Therefore, it may not be generalized to other healthcare setups. It also has limitations when compared to other countries due to healthcare setups, health policies, and other factors that are quite different among countries.

The other limitation of this evaluation was the hawthorn effect during observation of healthcare providers’ practices towards infection prevention. To minimize this bias, the observation was conducted by hospital staff.

## Conclusions

The evaluation result of this study concluded that the overall implementation fidelity of IPP was judged to be medium based on the judgment parameter and that it needs improvement. The implementation fidelity focused on adherence, participant responsiveness, and facilitation strategy dimensions.

The measurement of adherence was also judged to be medium, and the hospital had an IPC committee, an IPC team, and an IPC focal person with defined roles and responsibilities, but the IPC committee did not support the IPC team actively, and the facility leadership did not support the IPC program by allocating an adequate budget.

Participants’ responsiveness dimension was judged to be medium. It included client practices for infection prevention as well as client satisfaction with hospital cleanliness.

Clients’ infection prevention practice in the hospital was low. Client satisfaction with hospital cleanliness was high, accounting for approximately 82 percent. Admission to medical, gynaecology-obstetric, and orthopaedic wards and the educational level of clients were factors attributed to the satisfaction with the IPC program.

The measurement of the facilitation strategy was judged to be low, and it included IPC training that was delivered twice a year to all cleaning staff by the hospital. On the contrary, 21 percent of HCPs received the training in the first round, and around 3 percent of them received it in the second round. Amhara Regional Health Bureau better allocates an adequate budget for the infection prevention and control program, and the IPC program shall be considered during planning, budgeting, monitoring, and evaluation of activities, and the IPC program shall be incorporated into other facility routine activities. Debre Tabor Comprehensive Hospital is better able to deliver health education about infection prevention and control to clients to enable them to be sufficiently confident on their ability to involve their healthcare providers.

## Supplementary Information

Additional file 1.

## Data Availability

Data will be available upon request from the corresponding author.

## Abbreviations

AMR: Anti-Microbial Resistance
CASH: Clean and Safe Health facilities
CDC: Centers for Diseases Prevention and Control
DTCSH: Debre Tabor Comprehensive Specialized Hospital
EA: Evaluability Assessment
EFMOH: Ethiopian Federal Ministry of Health
IF: Implementation Fidelity
HAI: Health care Acquired Infection
HH: Hand Hygiene
IP: Infection Prevention
IPC: Infection Prevention and Control
IPPs: Infection Prevention practices
KII: Key Informant Interview
M&E: Monitoring and Evaluation
PPE: Personal Protective Equipment
SDG: Sustainable Development Goal
SPSS: Statistics Package for Social Sciences
SSI: Surgical Site Infection
WASH: Safe Water Sanitation and Hygiene
WHO: World Health Organization
